# Clarification of Limed Sugarcane Juice by Stainless Steel Membranes and Membrane Fouling Analysis

**DOI:** 10.3390/membranes12100910

**Published:** 2022-09-20

**Authors:** Nan Du, Lili Pan, Jidong Liu, Lijun Wang, Hong Li, Kai Li, Caifeng Xie, Fangxue Hang, Haiqin Lu, Wen Li

**Affiliations:** 1 College of Light Industry and Food Engineering, Guangxi University, Nanning 530004, China; 2 Engineering Center for Sugarcane and Canesugar, Guangxi University, Nanning 530004, China; 3 Collaborative Innovation Center of Guangxi Sugarcane Industry, Guangxi University, Nanning 530004, China

**Keywords:** stainless steel membrane, juice clarification, ultrafiltration, impurity removal, membrane fouling

## Abstract

The performance of stainless steel membranes with pore sizes of 100 and 20 nm in clarifying limed sugarcane juice was investigated under different operating conditions. An increase in transmembrane pressure (TMP) for the 20 nm membrane from 2 to 5 bar led to an increase in the average flux from 146.6 Lm^−2^ h^−1^ to 187.8 Lm^−2^ h^−1^ (approximately 9 h). The increase in crossflow velocity from 2 to 5 m/s led to an increase in the average flux from 111.9 Lm^−2^ h^−1^ to 158.1 Lm^−2^ h^−1^. The increase in temperature from 70 °C to 90 °C caused an increase in the average flux from 132.8 Lm^−2^ h^−1^ to 148.6 Lm^−2^ h^−1^. Simultaneously, the test produced a high-quality filtered juice with an average of 1.26 units of purity rise. The purity increased with time, and a 99.99% reduction in turbidity and an average 29.3% reduction in colour were observed. In addition, four classic filtration mathematical models and scanning electron microscopy (SEM) analyses suggested that cake formation is the main mechanism for flux decline. Fourier transform infrared (FTIR) spectrometry and energy-dispersive X-ray (EDX) spectrometry indicated that organic fouling is the main foulant. This study demonstrates the potential of stainless steel membranes as filters for the clarification of raw sugarcane juice.

## 1. Introduction

In sugar factories, ensuring the production of juice with consistently high clarity and low colour through clarification is a challenging task. The variations in the incoming juice characteristics, given the differences in sugarcane variety, soil and growing conditions, climate, and season, make this task extraordinarily difficult [1]. The three conventional methods of clarification are as follows: (1) the lime process, which produces raw sugar; (2) the double sulfitation process, which produces plantation white granulated sugar; and (3) the carbonation process, which produces high-quality plantation white granulated sugar [2]. These three conventional methods have numerous disadvantages, such as extensive processing, low product quality, large usage of chemicals, high energy costs, and environmental pollution [3]. The sugar industry should identify effective methods for clarifying raw sugarcane juice to improve its quality and reduce or eliminate the use of chemicals [4].

Recently, clarification and decolourisation via membrane filtration has gained importance, and membrane filtration applications in the food industry have become widely accepted because of their low energy consumption, high efficiency, and simplicity of operation [5,6,7]. Clarification of sugarcane juice via ultrafiltration (UF) is technically superior to the traditional separation process because UF yields juice that has high purity and improved colour quality and is free from starch and acidified substances [1,2,3,4,5,8,9]. Membrane technology has extensive application prospects in the sugar industry to address the aforementioned conventional clarification problems. Studies have shown that the membrane process improves the quality of clarified juice by removing colourants and enhancing purity [9,10,11,12]. The UF of the limed sugarcane juice that uses ceramic membranes showed more than 1.7 units of purity rise, a 99.6% reduction in turbidity, and a 38.9% reduction in colour [1]. Similarly, the purity increases by 4.38% and the colour is reduced by 96.55% with the use of an integrated polymeric membrane process [13]. However, some problems with the UF technology still need to be solved in practical applications.

First, it is difficult to meet the high permeate flux and clarification effect simultaneously, which requires a tradeoff [8]. In a pilot plant test, Li et al. observed an average permeate flux of 119.1–142.4 Lm^−2^ h^−1^ and purity increase over filtration of 1.2 units when the heated limed sugarcane juice was processed using a ceramic membrane [9]. Jegatheesan et al. produced filtered sugarcane juice with more than 1.7 units of purity rise, a 99.6% reduction in turbidity, and a 38.9% reduction in colour, but detected an average flux of only 35.2–65.6 Lm^−2^ h^−1^. Second, severe membrane fouling and flux decline occurred as the processing time increased, considering the suspended matter and complex composition of the raw mixed juice. Therefore, membrane fouling not only reduces the membrane flux, but also causes the membrane to change its decomposition and reduces its service life. Third, the membranes commonly used in sugarcane juice filtration have certain defects that require correction [8]. Currently, the most used ultrafiltration media in the sugar industry are ceramic and organic membranes. For ceramic membranes, expensive raw materials and the requirement of high sintering temperatures limit their application [10,11]. In addition, lower packing densities owing to the fragility of ceramic membranes are key challenges for operational conditions [12,14]. In addition, sugarcane juice should be heated to a high temperature (90 °C) to improve the flux and satisfy the requirements of evaporation; however, organic membranes are not resistant to high temperatures. Luo et al. determined that an operating temperature of 60 °C had a significant effect on the polyamide membrane performance [13]. The permeate flux of the UF membrane decreased to 10 Lm^−2^ h^−1^ at a high transmembrane pressure (TMP) of 10 bar. The metallic membrane exhibited acceptable hot-shock resistance, no temperature limitations, and suitability compared with those of ceramic and organic membranes [15,16,17]. Metallic membranes have thus been successfully used in the sugar industry.

The present study investigated the performance of a pilot plant-scale stainless-steel membrane system for treating limed sugarcane juice. This study aimed to (i) evaluate the flux obtained using membranes with different pore sizes under different operating conditions, (ii) evaluate the quality of permeate by the membrane, (iii) select an appropriate mathematical model to predict the performance of the membranes, and (iv) reveal the composition of foulants to determine the appropriate cleaning solution.

## 2. Materials and Methods

### 2.1. Sugarcane Juice and Chemicals

Raw mixed sugarcane juice was directly obtained from five successive milling units in a local sugar mill in Lincang, Yunnan Province, China. An 80-mesh curved stainless steel screen was used to remove large fibres and several suspended solid particles from the raw sugarcane juice during collection. To avoid microbial growth and sucrose conversion, the mixed juice was treated with lime milk under constant stirring to rapidly increase its pH from approximately 5.1–5.4 to 7.1 ± 0.2. Subsequently, the mixed juice was heated at the temperature according to the test scheme (70 °C/80 °C/90 °C) using saturated vapour (approximately 120 °C). The pretreated mixed juice was used as the feed for the experiments, and the main reagents in the experiment are listed in Table 1.

### 2.2. Membrane Modules and Equipment

Two types of stainless steel membranes with different diameters were selected for use in this study. The characteristics of the stainless steel membranes are listed in Table 2. A large membrane tube diameter is advantageous for processing juice with a high content of suspended matter.

**Figure 1:**
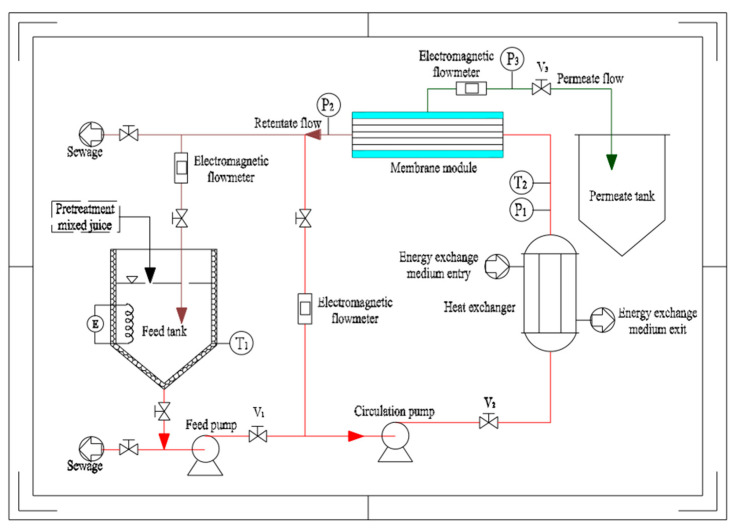
Schematic diagram of the 0.35 m^2^ stainless steel membrane experiment device.

The experimental setup for the system is illustrated in Figure 1. The pretreated mixed juice was continuously added to the feed tank kept at a constant volume of 40 L throughout the experiment. The temperature of sugarcane juice in the feed tank was maintained at the required temperature by electrical heating. Then, the juice was circulated through the heat exchanger and into the membrane module by the action of the feed and circulation pumps. The membrane tubes were welded to stainless steel constructions like the shell and tube-type heat exchangers. No seals or potting compounds presented chemical or temperature compatibility issues. The function of the heat exchanger is to remedy the temperature of the electric heating and compensate for the heat loss of the pipeline. The permeate was collected in the permeate tank and the retentate was recycled into the feed tank. The values V_1_ and V_2_ were adjusted to enable the membrane module to satisfy the experimental requirements, such as TMP and crossflow velocity (CFV). The pressure on the permeate side was adjusted to V_3_. Drain valves were provided at the highest and lowest points of the system. The three electromagnetic flowmeters monitored the direct circulation, total circulation, and permeate flows, respectively. The pressure, temperature, and flow data were displayed and adjusted using the computer of the system.

### 2.3. Experimental Procedure

The operating conditions of each experimental run are listed in Table 3. Each batch of raw mixed juice and samples from the feed and permeate tanks were collected at an initial and volumetric concentration factor of 1.5 and 3, respectively, for analyses. All experiments were conducted in a concentrated mode. The permeate was removed from the system and the retentate was recycled into the feed tank. Juice was drained from the system after each experiment. The membrane was rinsed with p water and then with chemicals. Finally, the membrane was rinsed with tap water. The water permeability was measured before and after feed filtration and cleaning.

### 2.4. Analytic Methods

All samples were analysed for purity, colour, turbidity, conductivity, ash content, and pH. The Standard International Commission for Uniform Methods of Sugar Analysis (ICUMSA) method was used for most index analyses. Turbidity was measured using a 2100 turbidimeter (HACH, Loveland, CO, USA). The colour concentration was determined by measuring the optical density of the solution at 560 nm using a HACH DR/2010 spectrophotometre, in accordance with the ICUMSA method. The SEM analysis was performed with scanning electron microscopy (Phenom Pro, Phenom, Rotterdam, The Netherlands). Information on the presence of specific functional groups on the fouled membrane surface was obtained using attenuated total reflection Fourier transform infrared (ATR-FTIR) spectroscopic imaging (Nicolet 50, Thermo Fisher Scientific, Waltham, MA, USA). The sample was dried prior to analysis. The energy-dispersive X-ray (EDX) analyser (PV8200, Philips, Amsterdam, The Netherlands) was used to identify organic fouling. The specimens were sputter-coated with gold.

### 2.5. Fouling Mechanism

To understand the mechanisms of flux decline, several researchers have proposed four classic filtration models to analyse the fouling mechanism. Four classic filtration models, that is, cake filtration, pore narrowing, a combination of external and progressive internal, and complete pore blocking, have been extensively adopted to interpret the fouling mechanism of membrane filtration of sugarcane juice under a constant pressure [18,19]. The fouling behavior has been successfully described using the Hermia models [20,21]. The corresponding equations are presented in Table 4. The experimental results were fitted in accordance with these equations, where the R-squared (R^2^) value indicated an acceptable fit.

## 3. Results

### 3.1. Flux

#### 3.1.1. Effect of Membrane Microstructure

**Figure 2:**
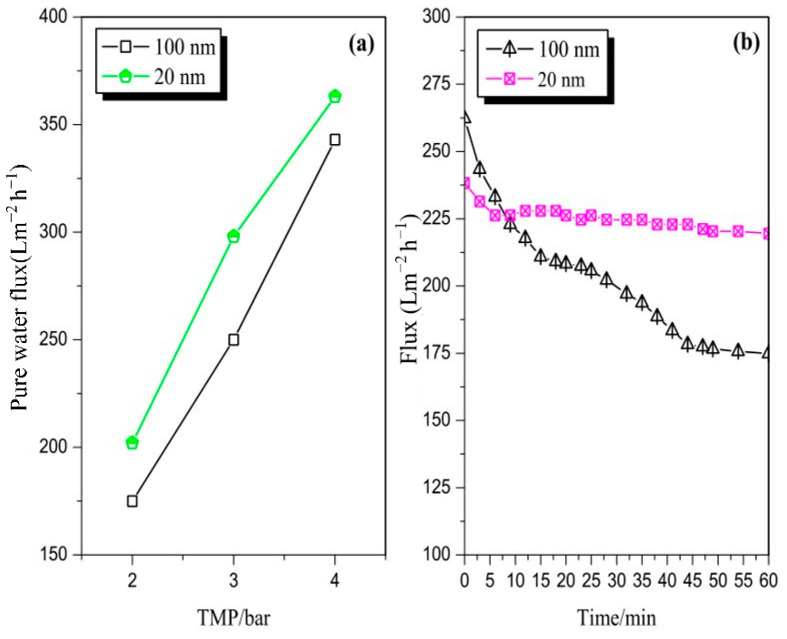
(**a**) The pure water flux comparison between 100 and 20 nm membranes under different operating conditions; (**b**) the flux of 100 and 20 nm membranes by filtered pretreated mixed juice (TMP of 3 bar, CFV of 3 m/s, and temperature of 70 °C).

Figure 2a illustrates the pure water flux comparison between the 100 and 20 nm membranes under different operating conditions. The flux increased with an increase in TMP. The flux of the 20 nm stainless steel membrane was higher than that of the 100 nm membrane under different test pressures (temperature of 20 °C). This result may be explained by the effect of the different porosity values of the two membranes given the different casting processes.

Figure 2b depicts the flux of the two pore sizes of the stainless steel membrane by filtered pretreated mixed juice at a TMP of 3 bar, CFV of 3 m/s, and temperature of 70 °C; the flux of 100 and 20 nm membranes was obtained in runs 1 and 2, respectively. A flux decline was observed in the first 9 min of the early stage of filtration. The flux of the 100 nm membrane was higher than that of the 20 nm membrane at this early stage. However, after the first 9 min, the flux of the 100 nm membrane was significantly lower than that of the 20 nm membrane. The flux of the 20 nm membrane dropped slightly at the beginning and then reached a stable state, thereby producing a steady-state flux of 225.0 Lm^−2^ h^−1^. The extent of flux decline was influenced by the membrane pore size. The flux difference between the two membranes may be caused by the size of the particles in the mixed juice that can cause the blockage of the 100 nm membrane pores [22]. These components led to fouling as the membrane pore and surface could be easily accessed. Notably, if the pore size is larger than the solute(s) size, then the possibility that the solute(s) can accesses the membrane pores is higher, thereby leading to a higher instance of pore narrowing than pore blocking. If the solute(s) size is larger than the pore size, then gel/cake layer fouling occurs. Thus, a large pore membrane does not automatically produce a high permeate flux. Other authors have also reported a similar phenomenon [23]. Hwang et al. [24] reported that large membrane pores are vulnerable to blockage and constriction, with significant effects on the flux. These effects are greater than that of the cake layer, which was formed on tight membranes.

#### 3.1.2. Effect of Operating Conditions

##### TMP

**Figure 3:**
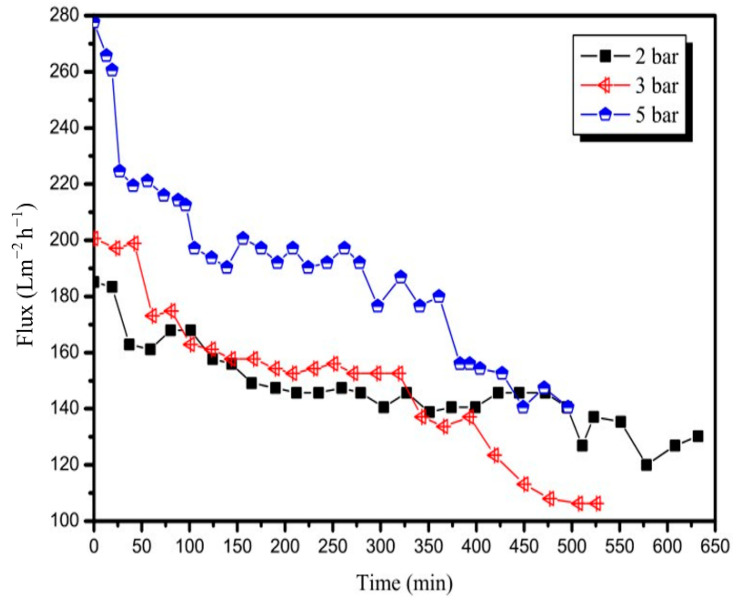
Variation in permeate flux with time for the 20 nm membrane at different TMPs (T = 90 °C, CFV = 3 m/s).

The effect of TMP on the permeate flux was studied using different TMP values, namely, 2, 3, and 5 bar, at a constant temperature of 90 °C and CFV of 3 m/s. Figure 3 shows the decline in the flux in the three runs. The initial fluxes obtained during the three runs using the 20 nm membrane were more than 180.0 Lm^−2^ h^−1^. The increase in TMP to 3 bar led to an increase in the initial flux from 185.1 Lm^−2^ h^−1^ (obtained by 2 bar) to 200.6 Lm^−2^ h^−1^, and the initial flux further increased to 277.7 Lm^−2^ h^−1^ when the TMP was increased to 5 bar. The average fluxes of the three runs were 146.6, 148.6, and 187.8 Lm^−2^ h^−1^. The decrease in the amplitude of the flux is thus affected by the TMP difference. The decrease in flux was rapid when the TMP was high. The decrease in the amplitude of flux obtained at TMP values of 3 and 5 bar was similar, but an increase of 38.1% in the average flux could be achieved when the TMP was increased from 3 to 5 bar. However, the average flux was similar when the TMP was increased from 2 to 3 bar, because the flux at 2 bar was relatively stable during the test, and the flux at 3 bar decreased rapidly after running for approximately 5 h. This behaviour implies that, initially, the filtration process can be controlled by pressure because the membrane surface is clean. As the filtration process continues, given the formation of a polarisation layer on the membrane surface, the filtration process becomes dependent on mass transfer. The effect of pressure on the flux becomes no longer significant.

##### CFV

**Figure 4:**
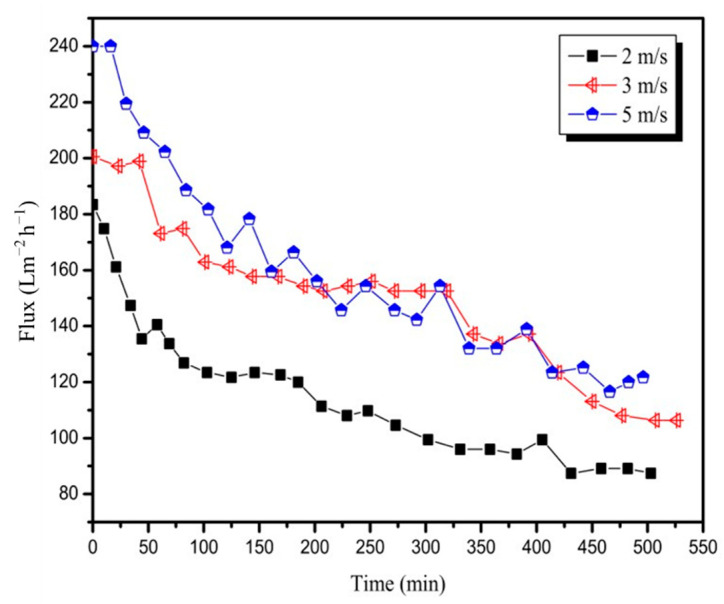
Variation in permeate flux with time for the 20 nm membrane at CFVs (T = 90 °C, TMP = 3 bar).

This study aimed to investigate the influence of CFV on flux in pretreated mixed juice. As shown in Figure 4, the initial flux increased with an increase in CFV. An increase in CFV from 2 to 5 m/s resulted in an increase in the initial flux from 183.4 Lm^−2^ h^−1^ to 240.0 Lm^−2^ h^−1^. This is because there was still space to store a certain volume of permeate between the membrane modules. As the membrane contamination increased, the flux decayed to different degrees, and the amount of accumulated permeate varied simultaneously. From the trend of flux decline with time, all flux declines caused by the 20 nm membrane at CFVs of 2, 3, and 5 m/s were significant, and the average fluxes of the three runs were 111.9, 148.6, and 158.1 Lm^−2^ h^−1^; the initial flux was reduced by approximately 50% at the end of the test. In addition, the smaller the CFV, the more serious the flux decay. At the beginning of filtration, the flux at 5 m/s of CFV was approximately 10% higher than the flux at 3 m/s of CFV owing to the increase in shear stress with an increase in CFV [25,26]. However, after 150 min of operation, as impurities in the juice accumulated on the membrane surface, the shear force was not sufficient to relieve the membrane fouling, and the flux at 5 m/s of CFV was similar to or even lower than that at 3 m/s of CFV.

##### Temperature

**Figure 5:**
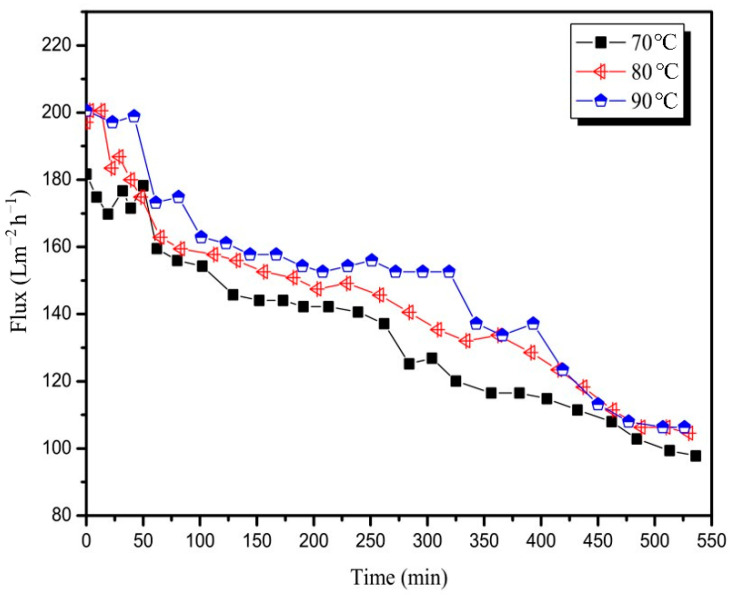
Variation in permeate flux with time for the 20 nm membrane at temperatures (TMP = 3 bar, CFV = 3 m/s).

The pretreated mixed juice was measured at a constant TMP of 3 bar and CFV of 3 m/s to examine the effect of the feed solution temperature on the permeate flux. As shown in Figure 5, the filtrate flux increased with an increase in temperature. This finding was consistent with the results of other filtering processes reported in the literature [27,28]. The feed viscosity decreased with an increase in temperature, and the molecular motion acceleration caused a reduction in the flow resistance, resulting in flux improvement. The average fluxes of the three runs were 132.8, 142.1, and 148.6 Lm^−2^ h^−1^.

### 3.2. Clarified Sugarcane Juice Quality

Table 5 summarises the typical quality parameters of the feed and permeate streams obtained in run 2. The permeate stream indicated that the average 1.26 units of purity increased, and the purity generally increased with time. A 99.99% reduction in turbidity and an average 29.3% reduction in colour were obtained. Like purity, the colour of the filtered permeate improved with time. However, the conductivity of the ash exhibited a consistent value. The purity of the retentate decreased, whereas the turbidity and colour increased. This phenomenon in the permeate was mostly caused by the removal of non-sugar components such as colourants, polysaccharides, colloids, and proteins [23]. Ultimately, the purity and colour of the permeate changed. Two probable interpretations of this phenomenon are as follows. First, previous literature reported that microporous gels formed during the filtration process exert further interception effects on impurities [7]; thus, additional non-sugar molecules are trapped. The second interpretation is associated with the formation of calcium phosphate. Few soluble phosphates were available in raw sugarcane juice when sodium hydroxide was added to the system. Phosphate is produced at an elevated temperature for a period of time; it can absorb impurities in sugarcane juice and reject the membrane.

### 3.3. Analysis of Membrane Fouling Models

#### 3.3.1. Effect of TMP

**Figure 6:**
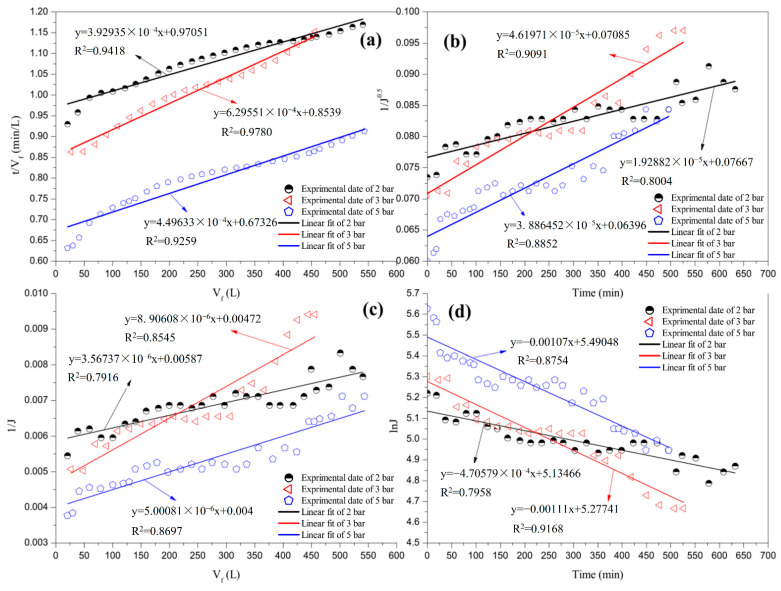
Linear fit with four fouling models at various TMPs: (**a**) cake filtration; (**b**) pore narrowing; (**c**) combination of external and progressive internal fouling; and (**d**) complete pore blocking.

Four classic filtration models were used to fit the experimental results to investigate the influence of TMP on the flux decline caused by pretreated mixed juice. Figure 6 and Table 6 present a comparison between the linear fit and the experimental data and the regression coefficients at different TMPs. The largest R^2^ values were 0.9418, 0.9780, and 0.9259, respectively, for the three runs. This result indicates that cake filtration fouling was the dominant phenomenon when the TMP was increased from 2 to 5 bar. A similar conclusion has been reported in previous studies [1,23]. It is also possible that the effect of pore narrowing was slightly involved, particularly under high-pressure conditions. The correlation coefficients of pore narrowing and complete pore blocking first increased and then decreased when the TMP increased from 2 to 5 bar. Furthermore, the correlation coefficients for the combination of external and progressive internal fouling increased. Colloid particles may not easily travel into the pores in the low operating pressure range because of the small pore size. Colloid particles were deposited instead on the membrane surface, resulting in cake fouling as the dominant phenomenon [25]. The linear correlation coefficients of the four classic filtration models were continuously strengthened with an increase in TMP from 2 to 3 bar. However, the pressure difference caused several small impurities to move through the cake layer and then transport through the pores when the TMP reached 5 bar. Cai et al. [29] stated that a high TMP increased the flow rate and shear force, leading to a reduction in the gel layer and a decrease in reversible fouling. The increase in shear force indicates that the force balance of the impurities deposited in the pore channel was off, thereby resulting in impurities that were separated from the channel into the permeate juice. Therefore, the linear correlation coefficients of cake filtration and pore narrowing decreased. Complete pore blocking exhibited a similar trend. This behaviour implies that pore blocking is significant under a low TMP, as reported by Hwang et al. [30]. Certain particles have a minimal opportunity to migrate to the membrane pores, considering the crowding produced by neighbouring particles when additional particles reach the membrane surface simultaneously. In contrast, particles have a high chance of penetrating pores if only a few particles accumulate on the membrane surface.

#### 3.3.2. Effect of CFV

**Figure 7:**
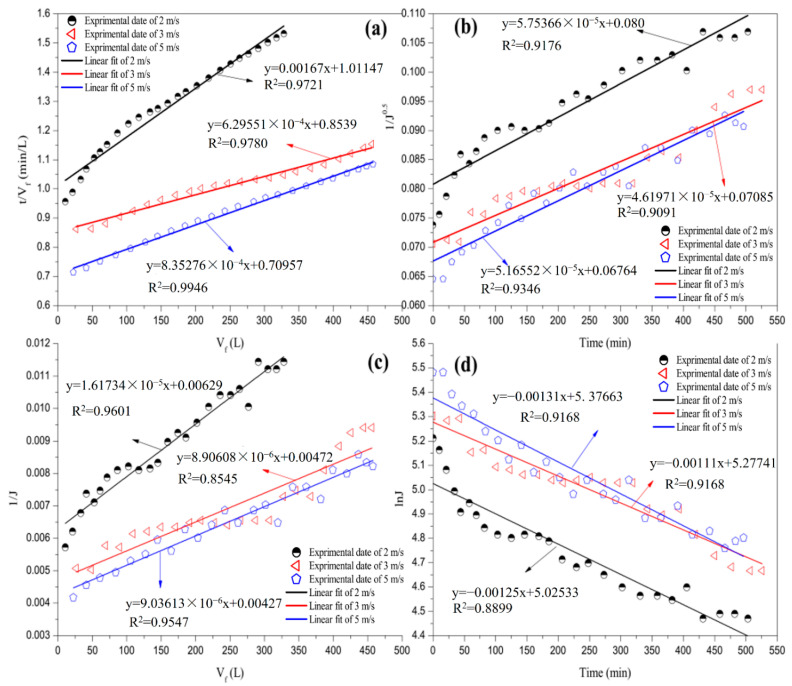
Linear fit with four fouling models at various CFVs: (**a**) cake filtration; (**b**) pore narrowing; (**c**) combination of external and progressive internal fouling; and (**d**) complete pore blocking.

Figure 7 and Table 7 display a comparison between the linear fit and the experimental data and the regression coefficients at different CFVs. As presented in Table 7, cake filtration fouling was the dominant phenomenon during the CFVs in the experiment, similar to the effect of TMP. The value of the regression coefficient for the cake filtration model increased as the CFV increased from 2 to 5 m/s. The combination of external and progressive internal fouling also played a crucial role in the formation of fouling in this study. As presented in Table 7, the regression coefficient of the combination of external and progressive internal fouling (R^2^ = 0.9601) was extremely close to that of the cake filtration (R^2^ = 0.9721) when the CFV was 2 m/s, and the combination of external and progressive internal fouling was the secondary dominant fouling mechanism. However, the R^2^ value for the combination of external and progressive internal fouling was the lowest when the CFV was 3 m/s. Again, the combination of external and progressive internal fouling became the secondary control of the filtration process with a continuously increasing CFV. These phenomena can be interpreted as the scenario wherein the shear force increases when the CFV increases [25,26]. The drag force of the fine particles in the low-CFV range was higher than the lift force. Thus, the small molecular impurities were deposited onto the membrane surface, thereby forming a fine filter cake layer. However, the large particles exhibit a high lift force. Thus, these particles did not come in contact with the layer or only formed a loose layer on the surface; this did not affect the membrane performance. The shear stress increased with an increase in the CFV. Several medium molecular impurities began to be deposited onto the membrane surface; this accelerated the formation of cake filtration and completed pore blocking when the CFV was 3 m/s. Therefore, the correlation coefficients for cake filtration and complete pore blocking increased. In contrast, several small molecular impurities, which were originally deposited on the pore channel, separated from the channel into the permeate juice given the large force. Therefore, the R^2^ values for pore narrowing and the combination of external and progressive internal fouling decreased. Large particles were deposited on the membrane surface, while the CFV continued to increase. Thus, the correlation coefficients of the four typical pollution models increased when CFV was 5 m/s, except for that of the complete pore blocking, which remained stable.

#### 3.3.3. Effect of Temperature

**Figure 8:**
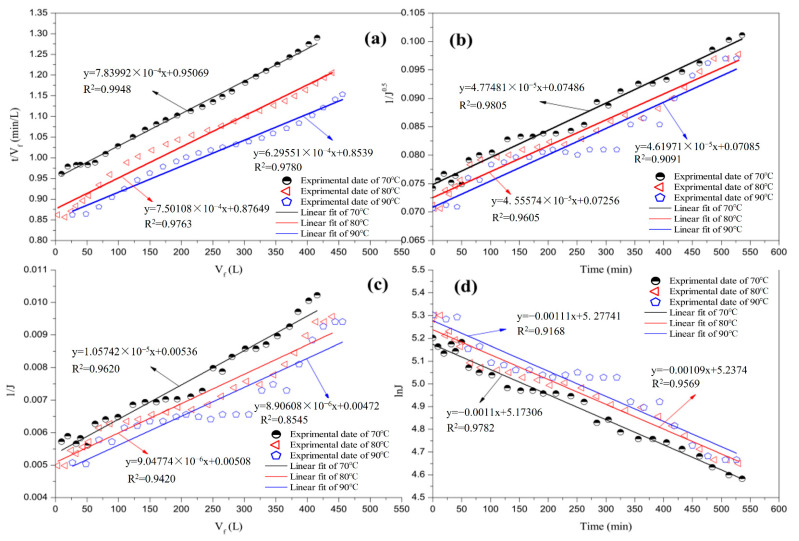
Linear fit with four fouling models at various temperatures: (**a**) cake filtration; (**b**) pore narrowing; (**c**) combination of external and progressive internal fouling; and (**d**) complete pore blocking.

Figure 8 illustrates the fitting of the different fouling models that correspond to the flux data of the entire filtration period during the test at different temperatures. Table 8 shows that cake filtration fouling remained the dominant phenomenon at different temperatures. The influence of temperature on the filtration process was complex. The viscosity of the juice decreased as the temperature increased. The diffusion coefficient then increased, thereby reducing the influence of concentration polarisation. Therefore, an increase in temperature promoted the reverse diffusion of foulants near the membrane surfaces to the main body of the juice. Certain foulants deposited on the membrane surface were returned to the juice system and the cake layer became thin. As presented in Table 8, the correlation coefficient of the cake decreased with the increase in temperature. However, the increase in temperature altered certain properties of the material; for example, there was a decrease in the calcium salt solubility in the cane juice, which subsequently increased the adsorption pollution, and the correlation coefficients of cake filtration and complete pore blocking at 80 °C declined. The correlation coefficient for the combination of external and progressive internal fouling also decreased. The continued increase in temperature significantly affected the forces on the membrane surface, pores, and impurities in the juice. Several particles that could not enter the membrane pore were able to move in on account of these changes. This phenomenon exacerbated the hole shrinkage at 90 °C.

### 3.4. Fouling Analysis

**Figure 9:**
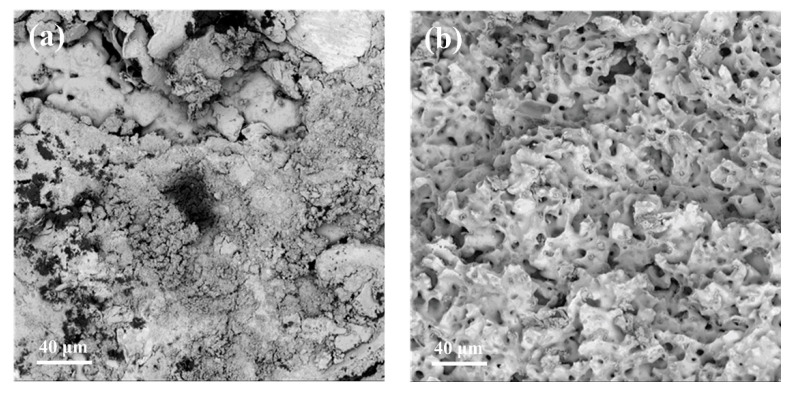
SEM micrographs of 20 nm fouled stainless steel membrane: (**a**) membrane layer (1000×); (**b**) support layer (1000×).

To further verify the membrane contamination, SEM was performed to observe the morphology of the contaminated 20 nm membrane. As illustrated in Figure 9a, the surface of the membrane was covered by a large amount of foulants owing to the presence of a large number of suspended substances in the sugarcane juice, whose particle size was larger than 20 nm. In contrast, in Figure 9b, the surface of the support layer shows a loose structure with dense pores, and no obvious contaminants are seen. The apparent height of the support layer varied owing to the uneven fracture. The SEM analysis showed that filter cake contamination played a key role in the stainless steel membrane separation of sugarcane juice.

**Figure 10:**
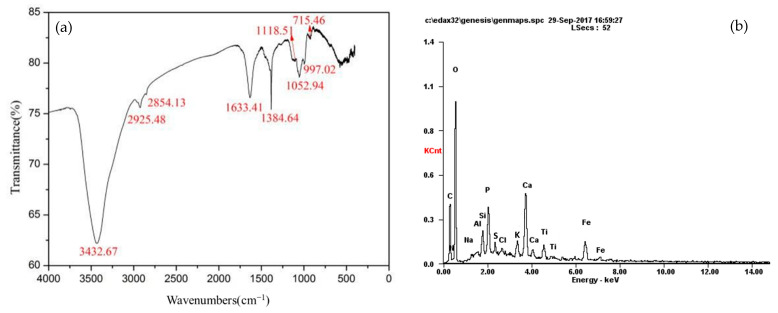
(**a**) FTIR spectra of foulants; (**b**) EDX spectra of foulants.

Figure 10a depicts the FTIR analysis results of the 20 nm stainless steel membrane after fouling with sugarcane juice. The spectrum indicated a specific peak at 3432.67 cm^−1^, which corresponded to the stretching and vibration of the O-H bond in the hydroxyl function groups [25,31,32]. Relatively high aliphatic CH_2_ absorption bands were observed at 2925.48 cm^−1^ (asymmetric stretching) and 2854.13 cm^−1^ (symmetric stretching) for organic matter [25,31,32,33]. The organic components deposited on the membrane surface were obtained from the sugarcane juice, which contains a carbon chain. The peak at 1633.41 cm^−1^ was attributed to the C=O stretching of the amide groups; this peak may have originated from protein substances in the sugarcane juice [25,31,32,33]. Two bands at 1384.64 (methyl stretching) and 1118.51 cm^−1^ (C-O stretching) were attributed to esters and phenols, respectively [25,31,32,33]. The bands at 1052.94 and 997.02 cm^−1^ were attributed to the C-O stretching of alcoholic compounds, which may have originated from polysaccharide-like substances [25,31]. The band at 715.46 cm^−1^ was attributed to the OH stretching vibration of carboxylic groups and deformation of COOH [25,31]. These results revealed that polysaccharides, esters, protein, phenols, and sucrose caused membrane fouling.

Elemental analysis was performed on the surface layer to identify the chemical components of the layer using EDX analysis. The EDX analysis of the membrane foulants in the sugarcane juice is presented in Figure 10b and Table 9. C and O are the major elements of foulants deposited on the membrane surface, and a certain amount of N is also observed. These results indicated that organic fouling played the most significant role as a foulant [13]. A minimal number of inorganic elements was observed in the pollutants (Al, Si, P, S, and K). These elements were extracted from the sugarcane juice. The Ca deposited onto the membrane surface was the result of adding minimal amounts of lime milk to adjust the pH of the juice prior to membrane UF. As presented in Table 9, minimal amounts of Fe were observed, which were mainly caused by the corrosion of the mill and tank by the acidic sugarcane juice during extraction. According to the investigations by Meng et al. [32] and Gao et al. [34], inorganic elements play a significant role in the formation of fouling layers, which could bridge the organic layer and form a dense cake layer when passing through the membrane, although the relative contents of these matters were minimal. The sharp Ti peak displayed in Figure 10b was caused by film peeling during the scraped foulants.

Determining the foulant components is beneficial for determining the cleaning solution. Inorganic and organic fouling based on the characteristics of the cake layer formed on the membrane surface and other studies can be avoided or limited by appropriate pretreatment and/or implementation of chemical cleaning.

## 4. Conclusions

The performance of stainless steel membranes with pore sizes of 20 and 100 nm in clarifying limed raw sugarcane juice was investigated under different operating conditions, and the following conclusions were drawn:

(1) The soft water flux was better in the 20 nm stainless steel membrane than in the 100 nm stainless steel membrane. The flux of the 20 nm stainless steel membrane also demonstrated excellent performance when handling sugarcane mixed juice.
(2) For the 20 nm membrane, the increase in TMP from 2 to 5 bar increased the initial flux from 185.1 Lm^−2^ h^−1^ to 277.7 Lm^−2^ h^−1^ (approximately 9 h) and increased the average flux from 146.6 Lm^−2^ h^−1^ to 187.8 Lm^−2^ h^−1^ (approximately 9 h). The increase in CFV from 2 to 5 m/s also increased the average flux from 111.9 Lm^−2^ h^−1^ to 158.1 Lm^−2^ h^−1^ (approximately 9 h). In addition, the increase in temperature from 70 °C to 90 °C increased the average flux from 132.8 Lm^−2^ h^−1^ to 148.6 Lm^−2^ h^−1^ (11.9% increase).
(3) The test produced high-quality filtered juice with an average of 1.26 units of purity rise, and the purity increased with time. A 99.99% reduction in turbidity and an average 29.3% reduction in colour were observed. The colour of the filtered juice improved with time, like the purity.
(4) Among the four fouling models used to fit the experimental data, the cake filtration model fitted the performance under all operating conditions. Thus, cake filtration fouling was the dominant phenomenon.
(5) The results of FTIR and EDX analyses showed that organic fouling played the most significant role as a foulant. The results revealed that polysaccharides, esters, proteins, phenols, and sucrose likely caused membrane fouling. Inorganic elements (e.g., Al, Si, P, S, and K) also played a key role in the formation of fouling layers. The determination of foulant components is thus beneficial for identifying the suitable cleaning solution.

These results are sufficiently encouraging and merit further investigation.

## Figures and Tables

**Table 1:** Main reagents.

Reagent	Specification	Manufacturer
Calcium oxide	AR	Shanghai Sinopharm Chemical Reagent Co., Ltd.
Lead acetate	AR	Guangzhou Gangxin Chemical Co., Ltd.
Sodium hydroxide	AR	Tianjin Bodi Chemical Co., Ltd.
Hydrochloric acid	AR	Sinopharm Chemical Reagent Co., Ltd.

**Table 2:** Characteristics of membranes.

Characteristics	Description
Manufacturer	Graver Technologies, LLC
Membrane type	Tubular
Membrane material	TiO_2_
Membrane support material	Porous 316 L stainless steel
Nominal pore size (nm)	100 nm, 20 nm
Length (cm)	1500
Number of channels	4
Channel diameter (mm)	18
Membrane area (m^2^)	0.35

**Table 3:** Operating conditions of the membrane modules.

Run No.	Membrane Pore Size (nm)	TMP (bar)	CFV (m/s)	Temperature (°C)
1	100	3.0	3	70
2	20	3.0	3	70
3	20	3.0	3	90
4	20	3.0	5	90
5	20	3.0	2	90
6	20	3.0	3	90
7	20	5.0	3	90
8	20	2.0	3	90
9	20	3.0	3	80
10	20	3.0	3	70

**Table 4:** Equations of the four classic filtration models.

Models	Equation
Cake filtration	$\frac{t}{V_{f}}=\frac{1}{Q_{0}}+K_{c}V_{f}$
Pore narrowing	$\frac{1}{J^{0.5}}=\frac{1}{J^{0.5}}+K_{n}t$
Combination of external and progressive internal	$\frac{1}{J}=K_{e}V_{f}+b$
Complete pore blocking	$lnJ=lnJ_{O}+K_{b}t$

Note: *t* is the filtration time (min), *V_f_* is the volume of filtered juice (L), *Q*_0_ is the initial flow rate of permeate (Lh^−1^), *K_c_* is the cake filtration constant, *J* is the permeate flux of time *t* (Lm^−2^ h^−1^), *J*_0_ is the initial permeate flux (Lm^−2^ h^−1^), *K_n_* is the pore narrowing constant, *K_e_* and *b* are the combination of external and progressive internal constants, and *K_b_* is the complete blocking constant.

**Table 5:** The typical quality parameters of feed and permeate streams obtained in run 2.

Material	Time (h)	pH	Purity (%)	Turbidity (NTU)	Color (IU_560_)	Conductivity Ash (%)
Feed	0	7.20	80.20	2657	1789	1.85
Filtered	0	7.17	81.20	1.00	1352	1.86
	0.5	7.12	81.37	1.02	1265	1.79
	1	7.04	81.81	0.94	1178	1.82
Retentate	1	7.12	78.10	/	1940	1.77

**Table 6:** R^2^ values of regression by different TMPs for four fouling models.

TMP/bar	Cake Filtration	Pore Narrowing	Combination of External and Progressive Internal Fouling	Complete Pore Blocking
2	0.9418	0.8004	0.7916	0.7958
3	0.9780	0.9091	0.8545	0.9168
5	0.9259	0.8852	0.8697	0.8754

**Table 7:** R^2^ values of regression by the different CFVs for four fouling models.

CFV/(m/s)	Cake Filtration	Pore Narrowing	Combination of External and Progressive Internal Fouling	Complete Pore Blocking
2	0.9721	0.9176	0.9601	0.8899
3	0.9780	0.9091	0.8545	0.9168
5	0.9946	0.9346	0.9547	0.9168

**Table 8:** R^2^ values of regression by different temperatures for four fouling models.

Temperature/°C	Cake Filtration	Pore Narrowing	Combination of External and Progressive Internal Fouling	Complete Pore Blocking
70	0.9948	0.9805	0.9620	0.9782
80	0.9763	0.9605	0.9420	0.9569
90	0.9780	0.9091	0.8545	0.9168

**Table 9:** EDX data of foulants.

Element	C	N	O	Al	Si	P	S	K	Ca	Ti	Fe
**Wt%**	28.57	2.65	43.06	0.33	1.64	3.89	0.89	1.81	8.31	2.41	6.44

## Data Availability

Not applicable.
